# Psychometric evaluation of the sleep hygiene index: a sample of patients with chronic pain

**DOI:** 10.1186/1477-7525-11-213

**Published:** 2013-12-22

**Authors:** Sungkun Cho, Gye-Seok Kim, Jang-Han Lee

**Affiliations:** 1 Department of Psychology, Chungnam National University, 99 Daehak-ro, Yuseong-gu, Daejeon 305-764, Korea; 2 Department of Business Administration, Sangji University, 660 Usan-dong Wonju-si Gangwon-do, Korea; 3 Department of Psychology, Chung-Ang University, 221 Heukseok-dong, Dongjak-gu, Seoul 156-756, Korea

**Keywords:** Sleep Hygiene Index, Sleep quality, Chronic pain, Psychometric properties

## Abstract

**Background:**

Sleep Hygiene Index (SHI) was designed to assess sleep hygiene. Although the SHI has shown adequate psychometric properties in a nonclinical sample, it has not been validated in a sample with chronic pain. Also, its factor structure, measurement error, and incremental validity over and above other factors affecting sleep quality have not been investigated in a nonclinical sample. Thus, this present study aimed to extend prior psychometric investigation of the SHI. Specifically, we evaluated the factor structure, measurement error, and incremental validity as well as the reliabilities and concurrent validity of the SHI in a sample with chronic pain.

**Methods:**

A total of 161 patients seeking treatment in a tertiary pain center located in Seoul, Korea participated. To explore the factor structure of the SHI, we performed an exploratory factor analysis using principal component with varimax. Cronbach’s alphas and intraclass correlation coefficients were computed to investigate internal consistency and 2-week test-retest stability of the SHI, respectively. Measurement error was estimated using standard error of measurement and minimum detectable change (MDC) of the SHI. For concurrent validity, Pearson correlations were calculated to examine the relations between the SHI and outcome measures including background variables. Also for incremental validity, a hierarchical multiple regression was performed in relation to sleep quality.

**Results:**

Results indicated that two-factor solution is most appropriate; sleep disturbing behavior and environment (B/E) and irregular sleep-wake schedule. Results also showed that the internal consistencies and test–retest stability estimates of the SHI were deemed acceptable. At the 95% confidence level, the MDCs were 5.75 for ‘sleep disturbing B/E,’ 3.65 for ‘irregular sleep-wake schedule,’ and 7.49 points for total. The SHI was significantly correlated with age, depression, pain-related anxiety, and sleep quality. Also, sleep quality was significantly predicted by the irregular sleep-wake schedule subscale of the SHI, over and above background variables, pain intensity, depression, pain-related anxiety.

**Conclusions:**

The SHI has the reliability, measurement error, and concurrent and incremental validity support for assessing sleep hygiene in a sample with chronic pain.

## Background

Poor sleep quality has been known as one of the most common complaints in patients with chronic pain (PCP) [1,2]. According to the National Sleep Foundation [3], approximately two-thirds of PCP suffer from poor sleep quality and their relative prevalence of poor sleep quality is four times that of the general population. Findings from earlier studies have indicated that poor sleep quality is linked to a wide range of deleterious outcomes, such as greater pain, emotional distress, and physical and psychosocial disability [4-6]. In particular, persistent pain can impair sleep quality and impaired sleep quality can also lower pain tolerance, which can result in amplified sensations of pain. These reciprocal causal relations often lead PCP to fall into a vicious cycle [7-9]. Given these, it is reasonable to assume that effective pain management should enhance sleep quality to break the vicious cycle before it produces even worse problems.

Poor sleep quality in PCP has been reported to correlate with pain [10] and emotional distress [10,11]. Other than these factors, poor sleep quality in PCP may be also associated with behavioral and environmental factors such as poor sleep habits (referred to as sleep hygiene) [12]. Sleep hygiene refers to the daily practices, habits, and environmental factors that are necessary for improving the quality of a night-time sleep [13]. As pain becomes prolonged and persistent, PCP is more likely to develop poor sleep hygiene. For example, it is not uncommon that PCP stay inactive and rest in bed most of the day and exhibit irregular sleep-wake schedules [14]. Although the role of sleep hygiene on sleep quality has not been established in PCP, sleep hygiene is one of the first things to check when they complain about poor sleep quality [15].

There are, to our knowledge, three primary instruments designed to assess sleep hygiene: the Sleep Hygiene Awareness and Practice Scale (SHAPS) [16], the Sleep Hygiene Self-Test (SHST) [17], and the Sleep Hygiene Index (SHI) [18]. The first two instruments have been found to have relatively low internal consistency (Chronbach’s alphas = .47 for the SHAPS and .54 for the SHST), compared to the SHI (Chronbach’s alpha = .66). Moreover, these two instruments appeared to be developed with absence of clear rationale for item selection [18], while the SHI was developed from the diagnostic criteria for inadequate sleep hygiene as defined in the International Classification of Sleep Disorders [19]. The SHI has shown moderate internal consistency and good 2-week test-retest stability (*r* = .71, *p* < .001) and been associated with sleep quality and daytime sleepiness in a nonclinical sample [18]. Although the SHI has shown adequate psychometric properties in a nonclinical sample, it has not been validated in a sample with chronic pain. Also, its factor structure, measurement error, and incremental validity over and above other factors affecting sleep quality have not been investigated in a nonclinical sample. Thus, this study aimed to 1) determine the factor structure of the SHI and 2) examine reliability, measurement error, and concurrent and incremental validity of the SHI over and above background variables, pain, and emotional distress (i.e., depression and pain-related anxiety) in PCP. As evidence for concurrent validity, poorer sleep hygiene is expected to significantly correlate with greater pain intensity, greater depression, greater pain-related anxiety, and lower sleep quality.

## Methods

### Participants

A total of 161 patients seeking treatment in a tertiary pain center located in Seoul, Korea participated. The sample was assessed at two time points, to examine test–retest stability over a 2-week interval of the SHI. After the patients completed an appointment at the pain center, volunteers were asked to complete the questionnaire packet in a private room. They also were provided with the identical questionnaire packet and a stamped addressed envelope for a second administration. They were instructed to take the packet to their home, complete and mail it back to the center after 2 weeks. One hundred thirteen patients (70%) completed the packet at time 2. Table 1 presents demographic characteristics of the sample. All data were collected and analyzed with approval by the Institutional Review Board (Seoul St. Mary’s Hospital) and informed consent by participants.

**Table 1:** Demographic characteristics of the sample

**Variable**	**Statistic**
Age (years)	
M	44.9
SD	14.0
Sex (%)	
Men	46.6
Women	53.4
Marital status (%)	
Married	66.5
Non-married	33.5
Education level (%)	
≥ High school	90.4
Pain duration (months)	
Median	36
Range	3-480
Taking pain-related medication (%)	65.2
Opioids	39.6
Non-opioids	60.4
Most significant pain site(s) (%)	
≥ 2 sites	45.2
Lower back	15.9
Leg(s)	7.6
Head	5.1
Foot(feet)	4.5
Others	21.7

### Translation

The two Korean speaking clinical psychologists (one who specialized in chronic pain and was aware of the objective of the SHI; the other was not) separately translated the SHI into Korean. Any inconsistencies were adjusted based on the agreement between them. Then, a Korean-English bilingual graduate student who had no knowledge of the SHI back-translated it into English. The back-translation version was reviewed by one of the original authors of the SHI and revised accordingly. The pre-final version of the SHI was tested by 5 patients with chronic pain. Each patient was asked to complete the SHI and to provide feedback regarding the wording and meaning of the each item of the SHI. Thus, the pre-final version of the SHI was finally revised based on their feedback.

### Measures

Demographic information was first obtained from a questionnaire. Items included age, sex, marital status, education level, and pain-related questions such as pain duration, pain-related medication, most significant pain site(s), and pain intensity. Pain intensity was measured by averaging present, usual, lowest, and highest pain during the past week, on a 0 (no pain) to 10 (worst possible pain) numeric rating scale (NRS). Total scores range from 0 to 10, with higher score representing worse pain. The NRS has been shown to have adequate reliability and validity in pain research [20].

The Sleep Hygiene Index (SHI) [18] is a 13-item self-report measure designed to assess the practice of sleep hygiene behaviors. Each item is rated on a five-point scale ranging from 0 (never) to 4 (always). Total scores range from 0 to 52, with a higher score representing poorer sleep hygiene. SHI has shown adequate reliability and validity [18].

The Center for Epidemiologic Studies Depressed Mood Scale (CES-D) [21] is a 20-item self-report measure designed to assess depressive symptoms. Each item is rated on a four-point scale ranging from 0 (rarely or none of the time) to 3 (most or all of the time). Total scores range from 0 to 60, with a higher score representing greater depression. This study used a Korean language version of the CES-D which has been shown to have adequate reliability and validity [22].

The Pain Anxiety Symptoms Scale-20 (PASS-20) [23] is a 20-item self-report measure designed to assess pain-related anxiety. Each item is rated on a six-point scale ranging from 0 (never) to 5 (always). Total scores range from 0 to 100, with a higher score representing greater pain-related anxiety. This study used a Korean version of the PASS-20 which has been shown to have adequate reliability and validity [24].

The Sleep Quality Scale (SQS) [25] is a 28-item self-report measure designed to assess sleep quality. Each item is rated on a four-point scale ranging from 0 (rarely) to 3 (almost always). Total scores range from 0 to 84, with a higher score representing lower sleep quality. This study used a Korean version of the SQS which has been shown to have adequate reliability and validity [25].

### Statistical analysis

The SPSS 18.0 for Windows software was used for the analyses. In order to explore the factor structure of the SHI, we performed an exploratory factor analysis (EFA) using principal component with varimax. Although rules of thumb regarding sample size for EFA have not yet been established, one common rule of thumb is a sample size of at least 100 [26] and another is a subject-to-item ratio of 10:1 [27]. Given this, the sample size (N = 161) of the present study appeared to be minimally acceptable. The number of factors was retained based on eigenvalues, scree test, and parallel analysis using the mean eigenvalues and 95th percentile eigenvalues. Using eigenvalues greater than 1 has been considered to be the least accurate method for factor retention and thus, this study also employed alternative methods such as scree test and parallel analysis [28]. Also, only items with a factor loading of .32 or greater were retained. Tabachnick and Fidell [29] recommended .32 for a minimum factor loading, indicating that there is approximately 10% overlap in variance with the other items in that factor. An item loaded less than .32 or a cross-loading item was not retained. The final derived factor solution was then used for subsequent reliability and validity analyses. Cronbach’s alphas and intraclass correlation coefficients (ICC (2, 1)) were computed to investigate internal consistency and 2-week test-retest stability of the SHI subscale and total scores, respectively. Measurement error was estimated using standard error of measurement (SEM) and minimum detectable change (MDC) of the SHI total and subscale scores. These were calculated by the following formulas: SEM = SD√(1-r), where SD is the baseline standard deviation of the measurement and *r* is the 2-week test-retest stability coefficient and MDC = 1.96*√2*SEM. For concurrent validity, Pearson correlations were calculated between the SHI and outcome measures including background variables. Dummy coding was used to represent a categorical variable (i.e., sex; male coded 0, female coded 1). Given the number of correlations, a *p* value was adjusted (i.e., *p* < .006) for more conservative prediction [30]. Also for the incremental validity of the SHI over and above background variables, pain intensity, depression, and pain-related anxiety, a hierarchical multiple regression was performed in relation to sleep quality (dependent variable). In each equation, participants’ age, sex, education, and pain duration were controlled first, and pain intensity, depression, and pain-related anxiety were controlled next, followed by the two subscales of the SHI entered in the final step.

## Results

EFA using principal component with varimax rotation was performed. The scree plot indicated that a 2-factor model was optimal. Both factors had eigenvalues greater than 1. The parallel analysis also indicated a 2-factor model and thus, 2-factor model was retained. All of the items were saliently loaded on either factor, except items 4 and 8. Specifically, item 4 was not saliently loaded on either factor and item 8 was saliently loaded on both factors. Thus, these items were excluded in the subsequent analyses. The 2-factor model accounted for 40.01% of the total variance (43.23% after excluding items 4 and 8) (Table 2), and the correlation coefficient among the factors was .31, *p* < .001 (.34 after excluding items 4 and 8), indicating a significant relationship among the factors. Considering the characteristics of the items loaded on each factor, factor 1 was labeled as ‘sleep disturbing behavior and environment (B/E)’ (e.g., I use alcohol, tobacco, or caffeine within 4 hours of going to bed or after going to bed) and factor 2 as ‘irregular sleep-wake schedule’ (e.g., I get out of bed at different times from day to day). Table 3 presents the factor loadings and item communalities of the 2-factor model.

**Table 2:** Forced two-factor solution by principal component with varimax rotation of items from the SHI

	**Initial eigenvalues**			**Extraction sums of squared loadings**			**Rotation sums of squared loadings**
**Component**	**Total**	**% of variance**	**Cumulative %**	**Total**	**% of variance**	**Cumulative %**	**Total**
1	3.33	25.59	25.59	3.33	25.59	25.59	2.65
2	1.88	14.42	40.01	1.88	14.42	40.14	2.55
3	1.19	9.15	49.17				
4	1.07	8.25	57.42				
5	.99	7.60	65.02				
6	.95	7.31	72.33				
7	.78	6.01	78.34				
8	.73	5.65	83.98				
9	.59	4.50	88.48				
10	.49	3.77	92.26				
11	.44	3.36	95.62				
12	.32	2.45	98.06				
13	.25	1.94	100.00				

Note: SHI: Sleep Hygiene Index.

**Table 3:** Two-factor solution: Factor loadings and communalities by principal components from the SHI

	**Factor loadings**		
**Item content**	**1**	**2**	***h***^***2***^
10. I sleep on an uncomfortable bed (for example: poor mattress or pillow, too much or not enough blankets).	**.77**	.06	.60
11. I sleep in an uncomfortable bedroom (for example: too bright, too stuffy, too hot, too cold, or too noisy).	**.74**	-.02	.55
12. I do important work before bedtime (for example: pay bills, schedule, or study).	**.65**	-.12	.43
7. I do something that may wake me up before bedtime (for example: play video games, use the internet, or clean).	**.51**	.23	.31
6. I use alcohol, tobacco, or caffeine within 4 hours of going to bed or after going to bed.	**.50**	.04	.25
13. I think, plan, or worry when I am in bed.	**.49**	.20	.28
9. I use my bed for things other than sleeping or sex (for example, watch television, read, eat, or study).	**.43**	.26	.25
4. I exercise to the point of sweating within 1 hour of going to bed.	.14	.06	.02
3. I get out of bed at different times from day to day.	.13	**.83**	.70
2. I go to bed at different times from day to day.	.13	**.82**	.69
5. I stay in bed longer than I should two or three times a week.	.06	**.74**	.55
1. I take daytime naps lasting two or more hours.	.00	**.50**	.25
8. I go to bed feeling stressed, angry, upset, or nervous.	**.34**	**.46**	.33

Note: Bold number indicates salient factor loading (≥.32); *h*^*2*^ indicates item communalities; SHI: Sleep Hygiene Index.

Table 4 presents descriptive statistics for the two subscale and total scores of the SHI and Table 5 presents the Cronbach’s α, ICC (2, 1), SEM, and MDC for the sleep disturbing B/E subscale, irregular sleep-wake schedule subscale, and total score. Table 6 presents descriptive statistics of the NRS, CES-D, PASS-20, and SQS. The data were all normally distributed except sex, education, and pain duration. However, given that Pearson’s correlation does not assume normality [31] and a non-parametric test (Spearman rank correlation) showed the similar results, we evaluated the relationship among all of the variables using Pearson’s *r*. Table 7 presents Pearson correlations between the SHI and outcome measures including background variables. Findings for background variables were mixed. Age was significantly negatively correlated with both subscales and total scores of the SHI, indicating younger individuals reported poorer sleep hygiene. Neither the subscale nor total scores of the SHI were significantly correlated with sex, education level, and pain duration. Findings for the four outcomes were also mixed, partly supporting the hypotheses. Greater depression and pain-related anxiety were significantly positively correlated with more irregular sleep-wake schedule and total score, but not sleep disturbing B/E. Sleep quality was significantly positively correlated with both subscales and total score of the SHI. Neither the subscale nor total scores of the SHI were significantly correlated with pain intensity. Incremental validity of the SHI over and above background variables, pain intensity, depression, and pain-related anxiety was further examined using hierarchical multiple regressions of the two subscales of the SHI in relation to sleep quality (dependent variable). The assumptions of multiple linear regression (i.e., linearity, independence of errors, homoscedasticity, multicollinearity, normality of errors) were satisfied. Generally, the finding indicated significant overall relation in the equation. Two subscales of the SHI added a significant increment in explained variance in the equation (ΔR^2^ = .02, *p* < .05). In the final step, the regression coefficients for irregular sleep-wake schedule (*β* = −.67, *p* < .05), but not for sleep disturbing B/E (*β* = .17, *n.s.*) were significant in the equation (Table 8).

**Table 4:** Descriptive statistics for subscale and total scores of the SHI

**Subscale**	**# items**	**Possible range**	**M**	**SD**	**Intercorrelations**	
					**1**	**2**
1. Sleep disturbing B/E	7	0-28	11.45	4.33		
2. Irregular sleep-wake schedule	4	0-16	7.70	3.21	.34^***^	
3. Total	11	0-44	19.16	6.58	.85^***^	.77^***^

Note: ***p < .001. SHI: Sleep Hygiene Index; B/E: behavior and environment.

**Table 5:** Internal consistency, 2-week test-retest reliability, and measurement error of the SHI

**Subscale**	**Chronbach’s α**	**ICC (2, 1) (95% CI)**	**SEM**	**MDC**
Sleep disturbing B/E	.74	.77 (.73-.89)	2.08	5.75
Irregular sleep-wake schedule	.70	.83 (.76-.88)	1.32	3.65
Total	.75	.83 (.73-.89)	2.71	7.49

Note: ICC: intraclass correlation coefficient; SEM: standard error of measurement; MDC: minimum detectable change; B/E: behavior and environment.

**Table 6:** Descriptive statistics for the NRS, CES-D, PASS-20, and SQS

	**Possible range**	**M**	**SD**
NRS (pain intensity)	0-10	5.31	2.31
CES-D	0-60	26.91	13.46
PASS-20	0-100	46.74	21.50
SQS	0-84	66.86	18.57

Note: NRS: Numerical Rating Scale; CES-D: Center for Epidemiologic Studies Depression Scale; PASS-20: Pain Anxiety Symptom Scale-20; SQS: Sleep Quality Scale.

**Table 7:** Correlations between the SHI subscale and total scores and background and outcome variables

	**Sleep disturbing B/E**	**Irregular sleep-wake schedule**	**Total score**
Age	-.26	-.38^*^	-.39^*^
Sex^a^	-.23	-.14	-.24
Education	.05	-.05	.01
Pain duration	-.08	-.03	-.07
Pain intensity	-.03	.15	.06
Depression	.12	.51^*^	.37^*^
Pain-related anxiety	.15	.46^*^	.35^*^
Sleep quality	.17	.52^*^	.41^*^

Note: *p < .006 adjusted p level of 0.006. ^a^male coded 0, female coded 1. SHI: Sleep Hygiene Index; B/E: behavior and environment. For concurrent validity, it was hypothesized that the SHI subscale and total scores are significantly positively correlated with four outcome variables (i.e., pain intensity, depression, pain-related anxiety, sleep quality).

**Table 8:** Results of hierarchical regression analyses predicting sleep quality from the two subscales of the SHI

**Step**	**Predictor**	***β *** **(Final)**	**ΔR**^**2**^	**Total R**^**2**^
1.	Age	-.01		
	Sex	.06		
	Education	−1.15		
	Pain duration	.00	.07^*^	
2.	Pain intensity	.14		
	Depression	.83^***^		
	Pain-related anxiety	.07	.55^***^	
3.	Sleep disturbing B/E	.17		
	Irregular sleep-wake schedule	-.67^*^	.02^*^	.63^***^

Note: *p < .05, ***p < .001. SHI: Sleep Hygiene Index; B/E: behavior and environment. Sex, education, and pain duration were not normally distributed. Thus, normality was further checked through histograms of the standardized residuals [32], and Q-plots and P-plots [33]. These tests supported the assumption of normality.

## Discussion

This present study aimed to extend a prior psychometric investigation of the SHI which assessed the reliabilities and concurrent validity in a nonclinical sample [18]. Specifically, we evaluated the factor structure, measurement error, and incremental validity as well as the reliabilities and concurrent validity of the SHI in a sample with chronic pain. Results from the present study indicated that two-factor solution is most appropriate, accounting for 43.23% of the variance. We interpreted the two factors as indicative of sleep disturbing B/E (7 items) and irregular sleep-wake schedule (4 items). Results also showed that the internal consistencies and test–retest stability estimates of the SHI subscale and total scores were deemed acceptable and relatively high compared to the prior study [18]. At the 95% confidence level, the MDCs were 5.75 for ‘sleep disturbing B/E’, 3.65 for ‘irregular sleep-wake schedule’, and 7.49 points for total. Changes over and above these points after intervention would likely not be measurement error.

Correlation analyses showed that age is negatively correlated with both subscales and total scores of the SHI. Overall, these findings suggest that younger people tend to show worse sleep hygiene. Depression and pain-related anxiety were positively correlated with the irregular sleep-wake schedule subscale and total scores of the SHI, suggesting that greater depression and pain-related anxiety are associated with worse sleep hygiene (especially regarding the aspect of irregular sleep-wake schedule). Both subscales and total scores of the SHI were positively correlated with sleep quality, suggesting that poorer sleep hygiene is associated with poorer sleep quality, which is consistent with prior studies [18]. Neither the subscale nor total scores of the SHI were significantly correlated with pain intensity. These findings suggest that pain may not be directly related to sleep hygiene. Instead, given that pain has been often considered to produce depression and pain-related anxiety [34], pain may be indirectly related to sleep hygiene. Further studies may benefit from examining possible interactions between pain intensity and depression and/or pain-related anxiety, in their relations with sleep hygiene.

In terms of incremental validity in relation to sleep quality, two subscales of the SHI added a significant increment in explained variance in the equation. Also, sleep quality was significantly predicted by the irregular sleep-wake schedule subscale only, over and above background variables and some primary factors affecting sleep quality (i.e., pain intensity, depression, pain-related anxiety) in PCP. This finding suggests the unique contribution of sleep hygiene to sleep quality and provides evidence for relative importance of sleep-wake schedule over sleep disturbing B/E in sleep quality of PCP. In fact, many PCP develop an irregular sleep-wake pattern through pharmacological and/or behavioral factors [35]. For example, PCP often take multiple medications and some of these (e.g., opioid and benzodiazepines) change their sleep-wake schedule. Also, many PCP fail to maintain a consistent daily activity pattern and spend considerable time in bed or inactive. More specifically, they may be in bed or inactive during episodes of severe pain. On the other hand, in response to some pain relief, they may overdo things that they were unable to do during the episodes of severe pain. This often leads to increases in pain followed by a few days of bed rest or inactivity for recovery. As a result, their sleep-wake schedule can be impaired, leading to poor sleep quality [35].

This present study has at least four limitations. First, although the process of translation/backtranslation was performed, it did not fully meet the guidelines for the process of cross-cultural adaptation of self-report measures [36]. Thus, items of the translated SHI may not be identical in meaning with those of the original SHI. Insufficient equivalence in meaning is more likely to limit the comparability of responses across different languages or cultures [36]. Second, although the sample size of this present study appeared to be minimally acceptable for EFA, a larger sample can help obtain a more stable solution [37] and increase generalizability to the population [38]. Third, this present study did not evaluate responsiveness of the SHI and cannot provide the criterion for clinically relevant change. Thus, responsiveness of the SHI scores to interventions should be evaluated in further research for the use of the SHI as a sign of changes in PCP. Finally, this present study employed a cross-sectional design and was composed of patients with diverse pain complaints, attending a tertiary pain center. Thus, causal relations among variables cannot be inferred and the findings may not generalize to other pain population (e.g., fibromyalgia, complex regional pain syndrome) or other settings (e.g., primary or secondary care clinic).

## Conclusions

The findings of this present study suggest that the SHI can be a useful research or clinical assessment tool for evaluating sleep hygiene to guide case formulation, treatment planning, or process analysis of intervention in pain centers. This present study provided evidence for the factor structure, reliability, measurement error, and concurrent and incremental validity of the SHI in PCP. However, given that this present study was the first attempt to validate the SHI in a sample with chronic pain, its application in clinical settings should come after further studies, including its utility in clinical decision support and aid in developing further treatment.

## Competing interests

The authors declare that they have no competing interests.

## Authors’ contributions

All of the authors have contributed in this study with design, data collection, and interpretation of the results. The manuscript was drafted by SC with supervision of GSK and JHL. All authors have read and corrected draft versions, and approved the final version.

## References

[B1] Hurley DA Eadie J O’Donoghue G Kelly C Lonsdale C Physiotherapy for sleep disturbance in chronic low back pain: a feasibility randomised controlled trial BMC Musculoskel Dis 2010 11 70 10.1186/1471-2474-11-70 PMC2873461 20398349

[B2] Menefee LA Cohen MJ Anderson WR Doghramiji K Frank ED Lee H Sleep disturbance and nonmalignant chronic pain: a comprehensive review of literature Pain Med 2000 11 156 172 10.1046/j.1526-4637.2000.00022.x 15101904

[B3] National Sleep Foundation Ask the sleep expert: pain and sleep http://www.sleepfoundation.org/article/ask-the-expert/pain-and-sleep

[B4] McCracken LM Iverson GL Disrupted sleep patterns and daily functioning in patients with chronic pain Pain Res Manag 2002 11 75 79 12185371 10.1155/2002/579425

[B5] Moldofsky H Sleep and pain Sleep Med Rev 2001 11 385 396 10.1053/smrv.2001.0179 12531004

[B6] Smith MT Haythornthwaite JA How do sleep disturbance and chronic pain inter-relate? Insights from the longitudinal and cognitive behavioral clinical trials literature Sleep Med Rev 2004 11 119 132 10.1016/S1087-0792(03)00044-3 15033151

[B7] Drewes AM Pain and sleep disturbances with special references to fibromyalgia and rheumatoid arthritis Rheumatology 1999 11 1035 1038 10.1093/rheumatology/38.11.1035 10556252

[B8] Drewes AM Arendt-Nielsen L Pain and sleep in medical diseases: interactions and treatment possibilities Sleep Res Online 2001 11 67 76

[B9] Kundermann B Krieg JC Schreiber W Lautenbacher S The effect of sleep deprivation on pain Pain Res Manag 2004 11 25 32 15007400 10.1155/2004/949187

[B10] Pilowsky I Crettenden I Townley M Sleep disturbance in pain clinic patients Pain 1985 11 27 33 10.1016/0304-3959(85)90227-1 4058926

[B11] Wilson KG Watson ST Currie SR Daily and ambulatory activity monitoring of sleep in patients with insomnia associated with chronic musculoskeletal pain Pain 1998 11 75 84 10.1016/S0304-3959(97)00207-8 9539676

[B12] Currie SR Wilson KG Pontefract AJ deLaplante L Cognitive–behavioral treatment of insomnia secondary to chronic pain J Consult Clin Psychol 2000 11 407 416 10883557 10.1037//0022-006x.68.3.407

[B13] Riedel BW Lichstein KL, Morin CM Sleep hygiene Treatment of of late-life insomnia 2000 Thousand Oaks, CA: Sage

[B14] Deardorff WW Practicing good sleep hygiene http://www.spine-health.com/wellness/sleep/practicing-good-sleep-hygiene

[B15] Perlis LM Cognitive behavioral treatment of insomnia 2005 New York, NY: Springer

[B16] Lacks P Rotert M Knowledge and practice of sleep hygiene techniques in insomniacs and good sleepers Behav Res Ther 1986 11 365 368 10.1016/0005-7967(86)90197-X 3729908

[B17] Blake DD Gomez MH A scale for assessing sleep hygiene: Preliminary data Psychol Rep 1998 11 1175 1178 10079712 10.2466/pr0.1998.83.3f.1175

[B18] Mastin DF Bryson J Corwyn R Assessment of sleep hygiene using the sleep hygiene index J Behav Med 2006 11 223 227 10.1007/s10865-006-9047-6 16557353

[B19] American Sleep Disorders Association International Classification of Sleep Disorders: Diagnostic and Coding Manual 1990 Rochester: MN: American Sleep Disorders Association

[B20] Jensen MP Karoly P Turk DC, Melzack R Self-report scales and procedures for assessing pain in adults Handbook of pain assessment 2001 New York, NY: Guilford

[B21] Radloff LS The CES-D scale A self-report depression scale for research in the general population Appl Psych Meas 1977 11 385 401 10.1177/014662167700100306

[B22] Chon KK Rhee MK Preliminary development of Korean version of CES-D Kor J Clin Psychol 1992 11 65 76

[B23] McCracken LM Dhingra L A short version of the Pain Anxiety Symptoms Scale (PASS-20): Preliminary development and validity Pain Res Manag 2002 11 45 50 16231066 10.1155/2002/517163

[B24] Cho S Lee SM McCracken LM Moon DE Heiby EM Psychometric properties of a Korean Version of the Pain Anxiety Symptoms Scale-20 in chronic pain patients Int J Behav Med 2010 11 108 117 10.1007/s12529-010-9080-2 20186509

[B25] Yi H Shin K Kim J Kim J Lee JB Shin C Validity and reliability of Sleep Quality Scale in subjects with obstructive sleep apnea syndrome J Psychosom Res 2009 11 85 88 10.1016/j.jpsychores.2008.07.008 19073298

[B26] Hair J Anderson RE Tatham RL Black WC Multivariate data analysis 1995 4 New Jersey: Prentice-Hall Inc

[B27] Cabrera-Nguyen P Author guidelines for reporting scale development and validation results in the Journal of the Society for Social Work and Research J Soc Work Res 2010 11 99 103 10.5243/jsswr.2010.8

[B28] Velicer WF Fava JL Effects of variable and subject sampling on factor pattern recovery Psychol Methods 1998 11 231 251

[B29] Tabachnick BG Fidell LS Using Multivariate Statistics 2001 Boston: Allyn and Bacon

[B30] Cano A Pain catastrophizing and social support in married individuals with chronic pain: the moderating role of pain duration Pain 2004 11 656 664 10.1016/j.pain.2004.05.004 15288406 PMC2396732

[B31] Rodgers JL Nicewander WA Thirteen ways to look at the correlation coefficient Am Stat 1988 11 59 66

[B32] Stevens JP Applied multivariate statistics for the social sciences 2009 5 New York, NY: Routledge

[B33] Keith T Multiple regression and beyond 2006 Boston, MA: Allyn & Bacon

[B34] Fisher BJ Cutler R Rosomoff HL Rosomoff RS Chronic pain associated with depression: Antecedent or consequence of chronic pain? A review Clin J Pain 1997 11 116 137 10.1097/00002508-199706000-00006 9186019

[B35] Cohen MJM Menefee LA Doghramji K Anderson WR Frank ED Sleep in chronic pain: problems and treatments Int Rev Psychiat 2000 11 115 126 10.1080/09540260050007435

[B36] Beaton D Bombardier C Guillemin F Ferraz M Guidelines for the process of cross-cultural adaptation of self-report measures Spine 2000 11 3186 3191 10.1097/00007632-200012150-00014 11124735

[B37] Cliff N The relation between sample and population characteristic vectors Psychometrika 1970 11 163 178 10.1007/BF02291261

[B38] MacCallum RC Widaman KF Zhang S Hong S Sample size in factor analysis Psychol Methods 1999 11 84 99

